# Tegafur/gimeracil/oteracil‐induced eosinophilic pneumonia

**DOI:** 10.1002/rcr2.551

**Published:** 2020-03-10

**Authors:** Toshiyuki Sumi, Hisashi Nakata, Yuji Mori, Hiroki Takahashi

**Affiliations:** ^1^ Department of Pulmonary Medicine Hakodate Goryoukaku Hospital Hakodate Japan; ^2^ Department of Respiratory Medicine and Allergology Sapporo Medical University School of Medicine Sapporo Japan

**Keywords:** Drug‐induced pneumonia, eosinophilic pneumonia, TS‐1

## Abstract

Clinicians should be aware that eosinophilic pneumonia is one of the types of drug‐induced lung injury that can be caused by tegafur/gimeracil/oteracil (TS‐1).

## Graphical abstract

We report a case of a 66‐year‐old man with drug‐induced eosinophilic pneumonia secondary to tegafur/gimeracil/oteracil (TS‐1) treatment for adenocarcinoma of the gastro‐oesophageal junction.

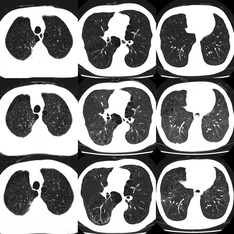

## Clinical Image

**Figure 1:**
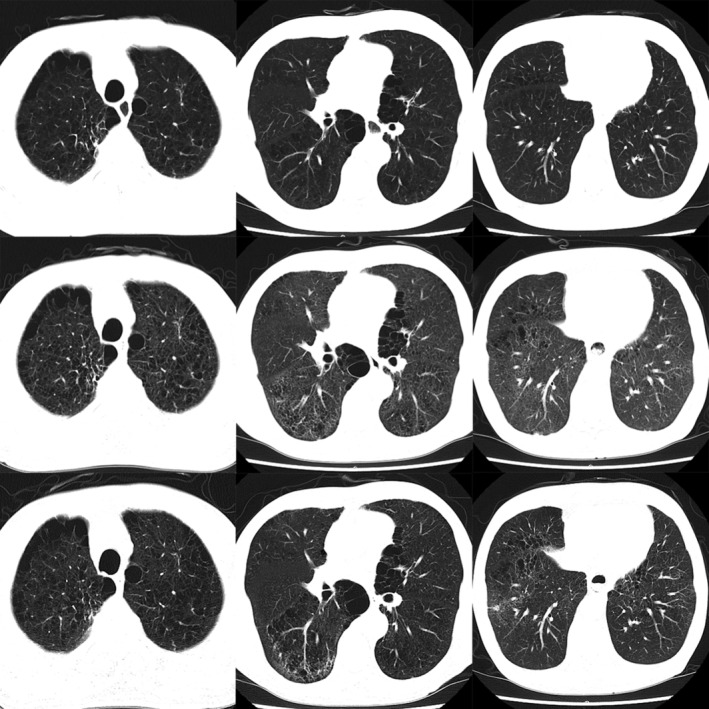
Chest computed tomography (CT) findings. Upper row is before the onset of symptoms, middle row is during presentation, and lower row is after 14 days of prednisolone therapy. Chest CT at onset revealed ground‐glass opacities in bilateral lung fields, which disappeared after prednisolone therapy.

**Figure 2:**
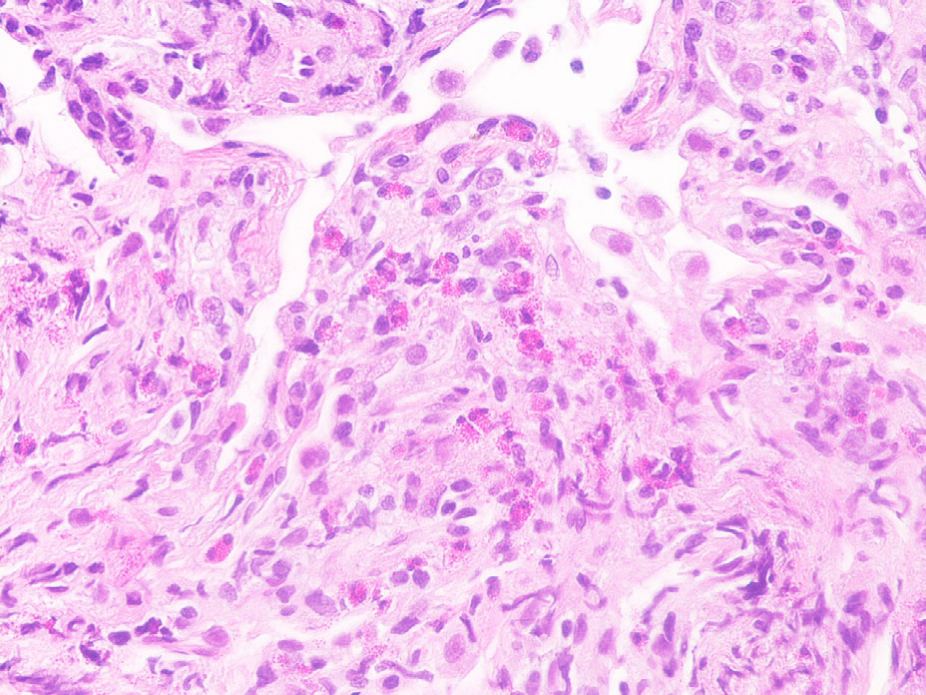
Histopathological findings following transbronchial lung biopsy. Lung specimens revealed fibrous thickening of the alveolar septum and eosinophil infiltration with eosinophilic granules (haematoxylin and eosin staining, 40×).

A 66‐year‐old man was admitted to the hospital with persistent high fever. He had received treatment with tegafur/gimeracil/oteracil (TS‐1) for adenocarcinoma of the gastro‐oesophageal junction 30 days previously. Chest computed tomography revealed ground‐glass opacities in bilateral lung fields (Fig. 1). Eosinophil fraction in peripheral blood had increased to 12.4% and that in the bronchoalveolar lavage fluid to 77%. Transbronchial lung biopsy revealed eosinophil infiltration in the lung tissues (Fig. 2), which suggested drug‐induced pulmonary toxicity; a drug lymphocyte stimulation test was highly positive for TS‐1. He was diagnosed with TS‐1‐induced eosinophilic pneumonia. Discontinuation of TS‐1 and initiation of prednisolone (starting from 0.5 mg/kg) improved his clinical symptoms and imaging findings (Fig. 1). Prednisolone was tapered over six weeks without recurrence. TS‐1, an oral drug with minimal side effects, is indicated in various malignancies. Drug‐induced pulmonary toxicity due to TS‐1 occurs rarely and there are a few reports. The histopathological findings commonly reported in TS‐1‐induced lung injury include organizing pneumonitis and non‐specific interstitial pneumonia 1, 2. To our knowledge, drug‐induced eosinophilic pneumonia secondary to TS‐1 has not been reported previously. Clinicians should be aware that eosinophilic pneumonia is a type of drug‐induced lung injury that can be caused by TS‐1.

### Disclosure Statement

Appropriate written informed consent was obtained for publication of this case report and accompanying images.
